# Cardioprotective effects of high-altitude adaptation in cardiac surgical patients: a retrospective cohort study with propensity score matching

**DOI:** 10.3389/fcvm.2024.1347552

**Published:** 2024-04-02

**Authors:** Li Lei, Mengxue Liu, Die Ma, Xia Lei, Si Zeng, Peng Li, Keli Huang, Juanjuan Lyu, Qian Lei

**Affiliations:** ^1^ Department of Anesthesiology, Sichuan Academy of Medical Sciences & Sichuan Provincial People’s Hospital, School of Medicine, University of Electronic Science and Technology of China, Chengdu, China; ^2^ Department of Cardiac Surgery, Sichuan Academy of Medical Sciences & Sichuan Provincial People’s Hospital, School of Medicine, University of Electronic Science and Technology of China, Chengdu, China; ^3^ Department of Pediatrics, West China Second University Hospital, Sichuan University, Chengdu, China

**Keywords:** high altitude, cardioprotective effect, cardiac surgery, major adverse events, ischemia-reperfusion injury

## Abstract

**Background:**

The cardioprotective effect of remote ischemia preconditioning in clinical studies is inconsistent with experimental results. Adaptation to high-altitude hypoxia has been reported to be cardioprotective in animal experiments. However, the clinical significance of the cardioprotective effect of high-altitude adaptation has not been demonstrated.

**Methods:**

A retrospective cohort study with propensity score matching was designed to compare the outcomes of cardiac surgery between highlanders and lowlanders in a tertiary teaching hospital. The data of adult cardiac surgical patients from January 2013 to December 2022, were collected for analysis. Patients with cardiopulmonary bypass and cardioplegia were divided into a low-altitude group (<1,500 m) and a high-altitude group (≥1,500 m) based on the altitude of their place of residence.

**Results:**

Of 3,020 patients, the majority (87.5%) permanently lived in low-altitude regions [495 (435, 688) m], and there were 379 patients (12.5%) in the high-altitude group [2,552 (1,862, 3,478) m]. The 377 highlander patients were matched with lowlander patients at a ratio of 1:1. The high-altitude group exhibited a 44.5% reduction in the incidence of major adverse cardiovascular events (MACEs) compared with the low-altitude group (6.6% vs. 11.9%, *P* = 0.017). The patients in the moderate high-altitude subgroup (2,500–3,500 m) had the lowest incidence (5.6%) of MACEs among the subgroups. The level of creatinine kinase muscle-brain isoenzymes on the first postoperative morning was lower in the high-altitude group than in the low-altitude group (66.5 [47.9, 89.0] U/L vs. 69.5 [49.3, 96.8] U/L, *P* = 0.003).

**Conclusions:**

High-altitude adaptation exhibits clinically significant cardioprotection in cardiac surgical patients.

## 1 Introduction

The environmental factor of altitude may be associated with the disparity in morbidity and mortality from cardiovascular diseases (1). However, the influence of altitude on the results of cardiac surgery is not well understood. Heart surgery-related intraoperative and postoperative organ damage is linked to a considerable increase in morbidity and mortality. Myocardial ischemia-reperfusion (I-R) injury during cardiopulmonary bypass (CPB) is an important cause of adverse outcomes after cardiac surgery. Clinical practice does not allow for the application of many short-term pretreatment techniques, such as hypoxic or myocardial ischemic preconditioning, which significantly reduce myocardial I-R injury in animal experiments (2). Remote ischemic preconditioning has also been confirmed to be cardioprotective in animal studies, nonetheless, the results of several clinical studies have generated controversy (3–7). In a multicenter randomized clinical trial involving adult patients underwent cardiac surgery, there was no significant difference in the incidences of primary end point between the remote ischemic preconditioning group (14.3%) and the control group (14.6%) (8). After 12 months following percutaneous coronary intervention, the occurrence rates of cardiac mortality or hospitalization due to heart failure were 8.6% in the control group and 9.4% in the remote ischemic conditioning group without statistical difference (3). Therefore, considerable attention has been drawn toward the organ-protective effect of chronic ischemia and hypoxia (9).

Individuals residing at high altitudes are exposed to a constant and powerful hypoxic environment. The long-term influence of adaptation to hypoxia at high altitudes has been a subject of interest in terms of its cardioprotective effect against acute I-R injury (10). Animal studies have demonstrated that adaptation to hypoxia can enhance the resistance to ventricular arrhythmias, contractile impairment, and myocardial infarction (11, 12). Interestingly, the signal pathway mechanisms of the cardioprotective effect exerted by prolonged hypoxia from high-altitude adaptation and short-term hypoxic or ischemic preconditioning appear to be similar (9, 13). However, the cardioprotective effect of high-altitude adaptation is longer than that of hypoxic or ischemic preconditioning. Additionally, chronic high-altitude hypoxia influences the expression of certain proteins involved in the maintenance of oxygen homeostasis (14, 15).

Several epidemiological studies have suggested that patients living in high-altitude regions may experience improved survival rates of coronary heart disease relative to individuals living in low-altitude regions (1, 16, 17). However, the issue related to myocardial I-R injury induced by CPB in patients born or living at plateau for a long period has not been comprehensively studied. There is a significant and sharp altitude drop from Qinghai-Tibet Plateau to the adjacent Sichuan Basin in southwest China. The aim of this study was to assess the influence of adaptation to high altitude on the clinical results following cardiac surgery according to medical data of patients from these regions.

## 2 Methods

### 2.1 Study design

Highlander patients from Qinghai-Tibet Plateau requiring major operations tend to seek treatment at several large teaching hospitals in the adjacent low-altitude regions. A retrospective cohort study with propensity score matching was designed to compare the perioperative data of cardiac surgery between highlanders and lowlanders. Patients from a single tertiary teaching hospital were enrolled into this study to avoid the confounding factor of different hospitals. After the Institutional Review Committee approval, the consecutive adult (age > 18 years) patients who had undergone cardiac surgery at Sichuan Provincial People's Hospital, located at a basin metropolis adjacent to the Qinghai-Tibet Plateau, China, from January 2013 to December 2022, were retrospectively reviewed. Exclusion criteria were as follows: (1) heart surgery without CPB; (2) beating-heart surgery under CPB without cardioplegia; (3) individuals born in regions with low altitudes but residing in regions with high altitudes, and vice versa; and (4) incomplete important electronic records, including primary end points and the data for propensity score matching. The Human Research Ethics Committee of the hospital approved the study (No. 2022–401), and the written informed consent was exempted. We used the STROBE guidelines for the design and reporting of the study.

### 2.2 Data sources and variables

All of the medical data for this study were retrieved from the big data system for clinical research, which was established from the electronic health record system and follow-up system of the hospital. The demographic information included places of residence and birth, age, gender, and body mass index (BMI). The following preoperative and intraoperative data were collected: principal diagnosis, comorbidities, cardiac function, routine preoperative examination results, history of cardiac surgery, principal cardiac procedures, CPB duration, and cross-clamp time. The patient's risk was calculated as EuroSCORE II (18).

The altitude of the patients' residence was obtained from Google Earth according to the community or village level of address. According to relevant studies and our previous report (16, 17, 19), patients living at an altitude < 1,500 m composed the low-altitude group, while those living at an altitude ≥ 1,500 m composed the high-altitude group. The high-altitude group was then divided into three subgroups, namely, mild high-altitude group (1,500 ≤ altitude < 2,500 m), moderate high-altitude group (2,500 ≤ altitude < 3,500 m) and very high-altitude group (altitude ≥ 3,500 m).

### 2.3 Outcomes

The primary outcome was the incidence of intraoperative or postoperative major adverse cardiovascular events (MACEs), defined as at least one of the following events happened in a patient: (1) myocardial infarction (MI) (defined as requiring percutaneous coronary intervention or redo operation for coronary artery bypass grafting); (2) cardiac arrest (defined as a postoperative event requiring cardiopulmonary resuscitation); or (3) low output syndrome or acute heart failure requiring extracorporeal membrane oxygenation (ECMO) or intra-aortic balloon pump (IABP) assistance. The secondary outcome measures were the incidence of in-hospital death and the level of creatinine kinase muscle-brain isoenzymes (CK-MB) on the first postoperative morning.

### 2.4 Statistical analysis and propensity score matching

The SPSS 27.0 software (IBM, USA) was utilized to analyze the data. The cohort database was initially reviewed to identify missing values. The proportion of missing data in different variables ranged from 0 to 7.3%. Patients with missing primary end points and data for propensity score matching were excluded (112/3132, 3.6%). Other missing continuous and categorical data were replaced by serial means and mode of the subgroup, respectively. Continuous variables were presented as mean ± standard deviation, or median (25th percentile, 75th percentile). Categorical variables were displayed as number (%). Continuous variables were compared using independent samples *t*-test and Wilcoxon rank-sum test. Categorical variables were analyzed using the *χ^2^* test or Fisher's exact test between the two groups. Propensity score matching was employed to minimize any potential discrepancies in the baseline characteristics. Each patient at high altitude was matched to one patient at low altitude according to their propensity score. The fixed caliper width was set at 0.02. The covariates included age, gender, BMI, left ventricular ejection fraction (LVEF), principal diagnosis, and EuroSCORE II. To explore the effect of difference altitude on the incidence of MACEs, a second order polynomial nonlinear regression analysis was performed to obtain a fitting curve among the different subgroups. A *P* value of lower than 0.05 was considered to be significant.

## 3 Results

### 3.1 Baseline characteristics

**Figure 1:**
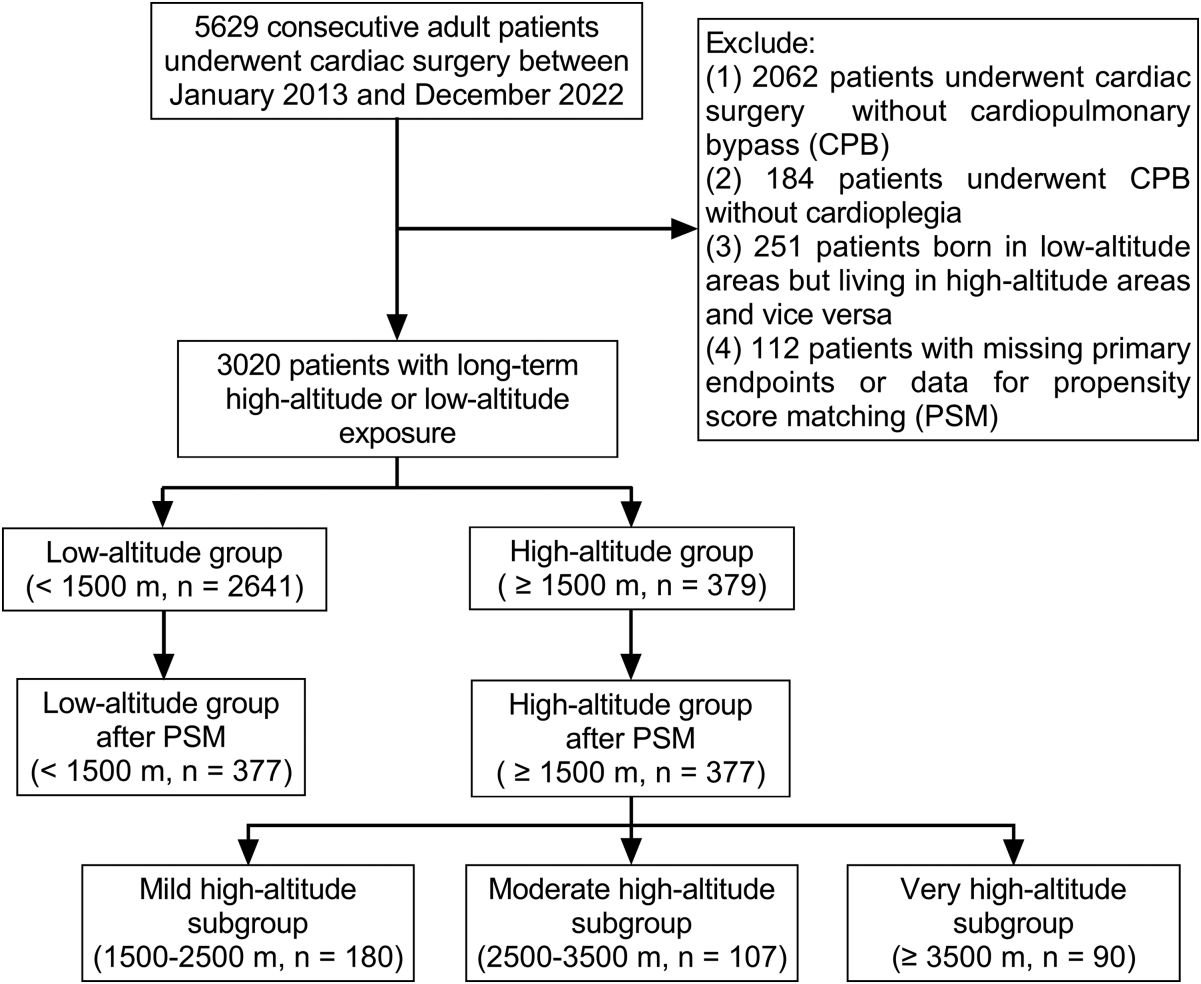
Study flowchart.

After excluding 2,609 patients, the initial analysis comprised 3,020 patients who had undergone cardiac surgery with CPB and cardioplegia (Figure 1). Most of the patients (87.5%) permanently lived in low-altitude regions [495 (435, 688) m above sea level], and there were 379 patients in the high-altitude group [2,552 (1,862, 3,478) m above sea level]. The highest inhabited elevation in the patients was 4,786 m. According to the range of altitudes, there were 180, 107, and 90 patients in the mild, moderate, and very high-altitude subgroups, respectively. The principal diagnosis with the largest proportion (42.8%) was valvular heart disease. According to the propensity score, 377 individuals from the low-altitude regions were matched at a 1:1 ratio with those from the high-altitude regions. The characteristics of the patients before and after propensity score matching are presented in Table 1. There were no significant intergroup differences in the baseline characteristics after matching.

**Table 1:** Analysis of demographic and clinical characteristics before and after propensity score matching.

Group	Before matching		After matching		Before matching		After matching	
	Low altitude (n = 2,641)	High altitude (n = 379)	Low altitude (n = 377)	High altitude (n = 377)	*P*-value	SMD	*P*-value	SMD
Demographic characteristics								
Age (years)	51.5 ± 12.3	46.7 ± 13.2	47.2 ± 13.1	46.7 ± 13.1	<0.001	−0.363	0.61	−0.038
Gender					0.014	0.139	0.23	0.011
Female	1,474 (55.8)	237 (62.5)	219 (58.1)	235 (62.3)				
Male	1,167 (44.2)	142 (37.5)	158 (41.9)	142 (37.7)				
BMI (kg/m^2^)	22.8 ± 3.4	23.0 ± 3.6	23.1 ± 3.5	23.0 ± 3.5	0.19	0.068	0.76	−0.022
Principal diagnosis								
Valvular heart disease	1,155 (43.7)	139 (36.7)	136 (36.1)	139 (36.9)	0.009	−0.146	0.82	0.016
Coronary artery disease	405 (15.3)	49 (12.9)	41 (10.9)	48 (12.7)	0.22	−0.072	0.43	0.055
Congenital heart disease	438 (16.6)	115 (30.3)	113 (30.0)	114 (30.2)	<0.001	0.299	0.88	0.006
Aortic disease	152 (5.7)	27 (7.1)	25 (5.8)	27 (7.2)	0.44	0.023	0.76	−0.091
Others	721 (27.3)	82 (21.6)	77 (20.4)	82 (21.8)	0.02	−0.137	0.66	0.032
LVEF (%)	62.6 ± 10.2	62.7 ± 9.0	62.6 ± 9.8	62.7 ± 9.0	0.89	0.009	0.32	0.013
Preoperative CK-MB (U/L)	11.1 (9.0, 14.6)	11.3 (9.0, 15.0)	11.0 (8.9, 14.0)	11.3 (9.0, 15.0)	0.21	0.032	0.13	−0.037
EuroSCORE II	1.76 ± 1.66	1.50 ± 1.45	1.48 ± 1.41	1.50 ± 1.46	0.004	−0.177	0.15	0.015
Operation duration (min)	314 ± 94	306 ± 83	313 ± 103	307 ± 83	0.14	−0.090	0.36	−0.075
CPB duration (min)	136 ± 57	124 ± 47	126 ± 54	124 ± 47	<0.001	−0.268	0.50	−0.053
Cross-clamp time (min)	91 ± 41	83 ± 37	84 ± 39	83 ± 37	<0.001	−0.228	0.75	−0.023

Data are displayed as mean ± standard deviation, median (25th percentile, 75th percentile), or number (%).

SMD, standardized mean difference; BMI, body mass index; LVEF, left ventricular ejection fraction; CK-MB, creatinine kinase muscle-brain isoenzymes; EuroSCORE II, European System for Cardiac Operative Risk Evaluation II.

### 3.2 Surgical data

The comparison of surgical data between the low- and high-altitude groups after propensity score matching is demonstrated in Table 2. There was no statistically significant disparity in the proportions of main procedures, CPB duration, and cross-clamp time.

**Table 2:** Comparison of surgical data between the low-altitude and high-altitude groups after propensity score matching.

Group	Low altitude (n = 377)	High altitude (n = 377)	*P*-value
Major procedures			
Aortic valve replacement	170 (45.1)	178 (47.2)	0.56
Mitral valve replacement	175 (46.4)	176 (46.7)	0.94
Mitral valve plasty	48 (12.7)	55 (14.6)	0.46
Tricuspid valve plasty	248 (66.1)	273 (72.4)	0.16
CABG	51 (13.5)	53 (14.1)	0.83
ASD repair	58 (15.4)	71 (18.8)	0.20
VSD repair	23 (6.1)	14 (3.7)	0.13
Aortic surgery	30 (8.0)	37 (9.8)	0.31

Data are displayed as number (%).

CABG, coronary artery bypass grafting; ASD, atrial septal defect; VSD, ventricular septal defect.

### 3.3 Patient outcomes

**Figure 2:**
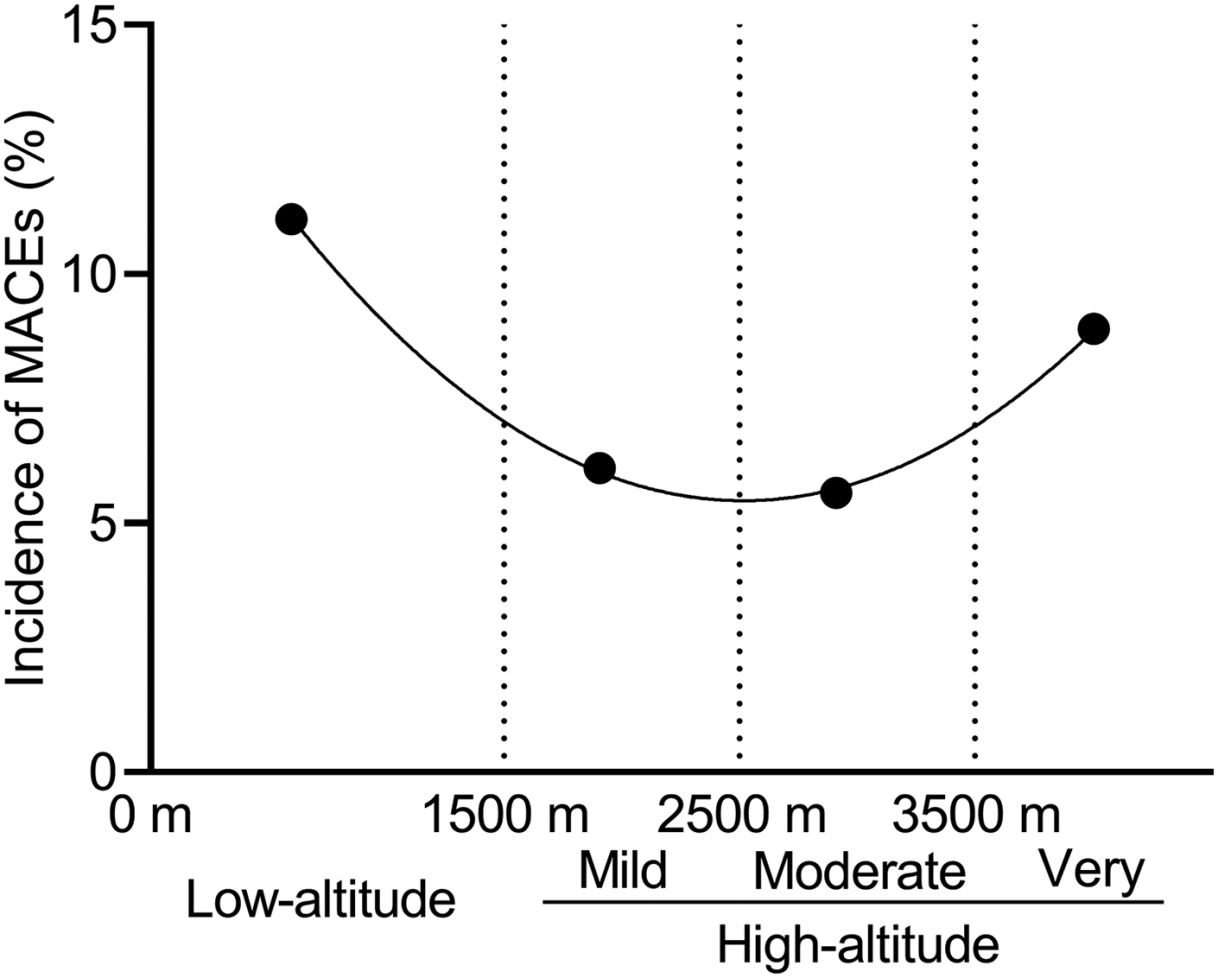
The incidence of major adverse cardiovascular events (MACEs) in different subgroups.

The comparison of the primary and secondary outcomes between the two groups is shown in Table 3. At least one MACE occurred in 45 of 377 patients (11.9%) in the low-altitude group and in only 25 of 377 patients (6.6%) in the high-altitude group after propensity score matching (*P* < 0.05). The incidence of intraoperative and postoperative MACEs in the high-altitude group was 44.5% lower than that in the low-altitude group. Most of the MACEs in both groups were IABP requirements (10.1% vs. 5.8%, *P* = 0.043). Only one of the patients in low-altitude group required ECMO support. There was a statistically significant difference in the occurrence of cardiac arrest between the two groups (*P* < 0.05). According to the fitting curve of the incidence of MACEs in the different subgroups, the patients living at altitudes of about 3,000 m had the lowest incidence of MACEs (Figure 2). The incidence of MACEs in the very high-altitude subgroup increased from 6.1% in the mild high-altitude subgroup and 5.6% in the moderate high-altitude subgroup to 8.9%.

**Table 3:** Comparison of outcomes of the low-altitude and high-altitude groups after propensity score matching.

Group	Low altitude (n = 377)	High altitude (n = 377)	*P* value	*Phi/Cohen d*
MACEs	45 (11.9)	25 (6.6)	0.017	0.190
Myocardial infarction	0 (0.0)	1 (0.3)	1.0	0.036
Cardiac arrest	18 (4.8)	6 (1.6)	0.021	0.191
ECMO	1 (0.3)	0 (0.0)	1.0	0.036
IABP	38 (10.1)	22 (5.8)	0.043	0.178
In-hospital death	12 (3.2)	5 (1.3)	0.14	0.063
Postoperative CK-MB (U/L)	69.5 (49.3, 96.8)	66.5 (47.9, 89.0)	0.003	44.4
MV time (h)	15.0 (8.0, 24.9)	14.8 (8.0, 26.1)	0.84	46.9
ICU length of stay (h)	42.0 (20.7, 88.6)	41.4 (21.0, 95.6)	0.60	95.1
In-hospital length of stay (d)	13.6 ± 9.7	13.7 ± 9.9	0.95	9.9

Data are displayed as mean ± standard deviation, median (25th percentile, 75th percentile), or number (%).

MACEs, major adverse cardiovascular events; IABP, intra-aortic balloon pump; ECMO, extracorporeal membrane oxygenation; CK-MB, creatinine kinase muscle-brain isoenzymes; MV, mechanical ventilation; ICU, intensive care unit.

The in-hospital mortality in the patients at high altitudes was lower than that in the patients at low altitudes (1.3% vs. 3.2%), however, there was no statistically significant difference (*P* = 0.14). The level of CK-MB on the first postoperative morning was significantly lower in the patients at high altitudes than in those at low altitudes (*P* < 0.05).

## 4 Discussion

### 4.1 Key results

To the best of our knowledge, this study represents the largest sample size so far in examining the effects of high-altitude adaptation on the clinical outcomes of cardiac surgical patients. Our findings indicate that chronic high-altitude adaptation is associated with a decrease in the occurrence of intraoperative and postoperative MACEs in cardiac surgery. The lower level of CK-MB on the first postoperative morning in the high-altitude group indicated an effect of myocardial protection from high-altitude adaptation as well. Patients who were chronically exposed to high altitudes showed a trend towards a lower rate of in-hospital mortality following cardiac surgery despite the absence of statistical significance. These findings provide strong evidence to confirm the clinical efficacy of adaptation to high-altitude hypoxia in myocardial protection (10).

### 4.2 High-altitude adaptation and myocardial protection

Significant progress has been made in the study of myocardial protection, but there is still a lack of effective prevention and treatment methods against myocardial I-R injury in clinical practice. For a considerable period, it has been recognized that transient hypoxic or ischemic preconditioning, remote ischemic preconditioning, and chronic hypoxia exposure offer protection to the heart against acute I-R injury in animals. Recent clinical trials have shown the limited improvement effect of transient limb remote ischemic preconditioning on clinical outcomes of surgical patients (3–7). Nonetheless, there are still doubts as to whether other hypoxic or ischemic methods can produce clinically significant myocardial protective effects. The presence of high-altitude hypoxia has been found to enhance the resilience to I-R injury in animal hearts, resulting in reduced myocardial infarct size and decreased occurrence and intensity of arrhythmias (15). High-altitude patients are natural and scarce human models for further exploring the clinical cardioprotective effects of adaptation to chronic hypoxia. The presence of clinical adverse events and myocardial injury indicators in highlanders suggests that the cardioprotection of chronic high-altitude hypoxia also applies to I-R injury induced by cardiac surgery under CPB. The obtained results are in agreement with previous experimental findings (20). While earlier studies primarily focused on ischemic heart disease, recent research has expanded to include other cardiovascular conditions (21). This study confirmed the clinical cardioprotective effect of adaptation to high-altitude hypoxia in cardiac surgical patients. Furthermore, the effect of adaptation to high altitude on myocardial protection was the best in the moderate high-altitude group. These results not only provide support for myocardial protection research related to ischemic or hypoxic preconditioning, but also provide a reference for high-altitude hypoxic adaptive activities, such as the altitude training of athletes.

### 4.3 Mechanisms underlying the cardioprotective effect of high-altitude adaptation

It is necessary to reveal the mechanism behind the cardioprotective effect of adaptation to high-altitude hypoxia, despite numerous experiments confirming its existence. Previous studies have shown that Tibetans who have lived at high altitudes for generations exhibit greater tolerance to hypoxia and experience fewer negative effects such as increased hemoglobin levels and pulmonary hypertension (22). In chronic hypoxia environments, individuals living at high altitudes undergo a metabolic adaptation to sustain their heart function. This adaptation results in a change in substrate preference, where their myocardial metabolism shifts towards utilizing glucose as the primary source when oxygen levels are reduced (23). The myocardial metabolic change can be used to explain the mechanism of cardioprotection against myocardial I-R in highlanders. The sympathetic system is activated in high altitude hypoxia environment (24). Chronic high-altitude hypoxia is known to elicit a cardioprotective effect, which is partly attributed to the presence of catecholamines. However, it has been demonstrated that the cardioprotective effect can be entirely nullified in a dog model of intermittent hypoxia by administering the β1 adrenergic receptor antagonist metoprolol prior to each hypoxic session. Additionally, it is worth noting that reactive oxygen species play a significant role in this process. Milano et al. (25) proposed that repeated reoxygenation may offer superior protection for the heart muscle compared with chronic hypoxia. Nevertheless, the duration of hypoxia experienced by individuals living in high-altitude regions is much longer. The long-term hypoxia exposure in highlanders may activate a mechanism that provides adequate protection for the heart muscle and other organs. Further, numerous studies have confirmed the protective effect of nitric oxide (NO) against I-R injury within the body (26). The severity of myocardial I-R injury can be diminished by administering NO or NO donors prior to the onset of heart ischemia. This intervention effectively reduces the size of the myocardial infarct and mitigates endothelial dysfunction. Interestingly, Tibetans exhibit elevated levels of NO in their circulation, which aids in enhancing oxygen delivery to cells when coupled with increased ventilation (22). However, it is imperative to thoroughly investigate the mechanisms behind the organ-protective effects that are triggered by environmental adaptation to high altitude in the forthcoming research. Additionally, chronic high-altitude hypoxia influences the expression of certain proteins involved in the maintenance of oxygen homeostasis (14, 15).

It has been suggested that the signaling pathways are similar for short-term myocardial ischemia, hypoxic preconditioning, and remote ischemic preconditioning to exert their myocardial protective effects. However, the prevention and treatment methods adopted for these mechanisms are difficult to improve the clinical outcomes of patients. Long-term adaptation to high altitude and low oxygen can produce a clear myocardial protective effect that improves clinical outcomes in patients. The mechanisms of this effect may somewhat differ between short-term myocardial ischemia, hypoxic preconditioning, and distant ischemic preconditioning. Although adaptation to high-altitude hypoxia is difficult to implement in clinical practice, elucidating its mechanism can lay the foundation for finding better clinical myocardial protection strategies and targets.

### 4.4 Limitations

There are several limitations to this study. First, some variables of cardiac complications were not collected for analysis because of uncertain data during the retrospective collection. Therefore, we chose clear definitions of end points for MACEs as the clinical indicator of myocardial injury. Furthermore, we discovered that high-altitude adaptation can lead to enhanced surgical results following cardiac surgery, however, the exact mechanism has not been fully understood. Further study in human samples of myocardium or blood might help to understand the possible mechanisms.

## 5 Conclusion

Individuals adapted to high altitude exhibit improved clinical outcomes, such as lower incidence of intraoperative and postoperative MACEs and lower level of postoperative CK-MB in patients undergoing cardiac surgery. These findings indicate that environmental adaptation to high altitude could potentially have a positive impact on minimizing myocardial I-R injury in cardiac surgical patients.

## Data Availability

The raw data supporting the conclusions of this article will be made available by the authors, without undue reservation.
