# Supplementary material for: Cardioprotective effects of high-altitude adaptation in cardiac surgical patients: a retrospective cohort study with propensity score matching
Source: Front Cardiovasc Med. 2024 Apr 2;11:1347552. doi: 10.3389/fcvm.2024.1347552 (PMC11019029; doi:10.3389/fcvm.2024.1347552)
Supplement: Supplementary file 1 [file Table1.pdf]

Table S1

Analysis of demographic and clinical characteristics before and after propensity score matching.

Group	Before matching		After matching		Before matching		After matching	
	Low altitude (n = 2641)	High altitude (n = 379)	Low altitude (n = 751)	High altitude (n = 377)	*P* value	SMD	*P* value	SMD
Demographic characteristics								
Age (years)	51.5 ± 12.3	46.7 ± 13.2	46.5 ± 13.2	46.7 ± 13.1	< 0.001	-0.363	0.89	0.014
Gender					0.014	0.139	0.78	0.019
Female	1474 (55.8)	237 (62.5)	461 (61.4)	235 (62.3)				
Male	1167 (44.2)	142 (37.5)	290 (38.6)	142 (37.7)				
BMI (kg/m^2)	22.8 ± 3.4	23.0 ± 3.6	22.9 ± 3.6	23.0 ± 3.5	0.19	0.068	0.74	0.023
Principal diagnosis								
Valvular heart disease	1155 (43.7)	139 (36.7)	261 (34.8)	139 (36.9)	0.009	-0.146	0.48	0.047
Coronary artery disease	405 (15.3)	49 (12.9)	93 (12.4)	48 (12.7)	0.22	-0.072	0.87	0.012
Congenital heart disease	438 (16.6)	115 (30.3)	220 (29.3)	114 (30.2)	<0.001	0.299	0.74	0.017

Aortic disease	152 (5.7)	27 (7.1)	47 (6.3)	27 (7.2)	0.44	0.023	0.22	-0.059
Others	721 (27.3)	82 (21.6)	182 (24.3)	82 (21.8)	0.02	-0.137	0.35	0.058
LVEF (%)	62.6 ± 10.2	62.7 ± 9.0	62.7 ± 10.0	62.7 ± 9.0	0.89	0.009	0.99	-0.001
Preoperative CK-MB (U/L)	11.1 (9.0, 14.6)	11.3 (9.0, 15.0)	11.1 (9.0, 14.0)	11.3 (9.0, 15.0)	0.21	0.032	0.41	-0.017
EuroSCORE II	1.76 ± 1.66	1.50 ± 1.45	1.58 ± 1.46	1.50 ± 1.46	0.004	-0.177	0.40	-0.051
Operation duration (min)	314 ± 94	306 ± 83	309 ± 98	307 ± 83	0.14	-0.090	0.70	-0.034
CPB duration (min)	136 ± 57	124 ± 47	125 ± 52	124 ± 47	<0.001	-0.268	0.76	-0.017
Cross-clamp time (min)	91 ± 41	83 ± 37	83 ± 39	83 ± 37	<0.001	-0.228	0.97	-0.002

Data are displayed as mean ± standard deviation, median (25th percentile, 75th percentile), or number (%). Abbreviations: SMD, standardized mean difference; BMI, body mass index; LVEF, left ventricular ejection fraction; CK-MB, creatinine kinase muscle-brain isoenzymes; EuroSCORE II, European System for Cardiac Operative Risk Evaluation II.
