# Supplementary material for: Cardioprotective effects of high-altitude adaptation in cardiac surgical patients: a retrospective cohort study with propensity score matching
Source: Front Cardiovasc Med. 2024 Apr 2;11:1347552. doi: 10.3389/fcvm.2024.1347552 (PMC11019029; doi:10.3389/fcvm.2024.1347552)
Supplement: Supplementary file 2 [file Table2.pdf]

Table S2

Comparison of surgical data between the low-altitude and high-altitude groups after propensity score matching.

Group	Low altitude<br>(n = 751)	High altitude<br>(n = 377)	*P* value
Major procedures			
Aortic valve replacement	324 (43.1)	178 (47.2)	0.19
Mitral valve replacement	323 (43.0)	176 (46.7)	0.24
Mitral valve plasty	99 (13.2)	55 (14.6)	0.52
Tricuspid valve plasty	505 (67.2)	273 (72.4)	0.078
CABG	109 (14.5)	53 (14.1)	0.84
ASD repair	112 (14.9)	71 (18.8)	0.092
VSD repair	47 (6.3)	14 (3.7)	0.075
Aortic surgery	53 (7.1)	37 (9.8)	0.11

Data are displayed as number (%). Abbreviations: CABG, coronary artery bypass grafting; ASD, atrial septal defect; VSD, ventricular septal defect.
