# Supplementary material for: Cardioprotective effects of high-altitude adaptation in cardiac surgical patients: a retrospective cohort study with propensity score matching
Source: Front Cardiovasc Med. 2024 Apr 2;11:1347552. doi: 10.3389/fcvm.2024.1347552 (PMC11019029; doi:10.3389/fcvm.2024.1347552)
Supplement: Supplementary file 3 [file Table3.pdf]

Table S3

Comparison of outcomes of the low-altitude and high-altitude groups after propensity score matching.

Group	Low altitude (n = 751)	High altitude (n = 377)	*P* value	Phi/Cohen d
MACEs	90 (12.0)	25 (6.6)	0.005	0.183
Myocardial infarction	2 (0.3)	1 (0.3)	1.0	0.00
Cardiac arrest	34 (4.5)	6 (1.6)	0.012	0.175
ECMO	2 (0.3)	0 (0.0)	0.32	0.030
IABP	75 (10.0)	22 (5.8)	0.019	0.170
In-hospital death	19 (2.5)	5 (1.3)	0.19	0.039
Postoperative CK-MB (U/L)	71.0 (50.4, 95.8)	66.5 (47.9, 89.0)	0.003	42.4
MV time	14.6 (7.5, 24.5)	14.8 (8.0, 26.1)	0.91	45.8
ICU length of stay	38.8 (20.5, 87.3)	41.4 (21.0, 95.6)	0.44	80.5
In-hospital length of stay	13.4 ± 9.3	13.7 ± 9.9	0.33	9.5

Data are displayed as mean ± standard deviation, median (25th percentile, 75th percentile), or number (%). Abbreviations: MACEs, major adverse cardiovascular events; IABP, intra-aortic balloon pump; ECMO, extracorporeal membrane oxygenation; CK-MB, creatinine kinase muscle-brain isoenzymes; CPB, cardiopulmonary bypass; ICU, intensive care unit.
